# Taking HIV Testing to Families: Designing a Family-Based Intervention to Facilitate HIV Testing, Disclosure, and Intergenerational Communication

**DOI:** 10.3389/fpubh.2016.00154

**Published:** 2016-08-05

**Authors:** Heidi van Rooyen, Zaynab Essack, Tamsen Rochat, Daniel Wight, Lucia Knight, Ruth Bland, Connie Celum

**Affiliations:** ^1^Human and Social Development Program, Human Sciences Research Council, Pietermaritzburg, South Africa; ^2^School of Law, University of KwaZulu-Natal, Pietermaritzburg, South Africa; ^3^Developmental Pathways to Health Research Unit, School of Clinical Medicine, University of Witwatersrand, Johannesburg, South Africa; ^4^Section of Child of Adolescent Psychiatry, Department of Psychiatry, Oxford University, Oxford, UK; ^5^MRC/CSO Social and Public Health Sciences Unit, University of Glasgow, Glasgow, UK; ^6^School of Public Health, University of the Western Cape, Cape Town, South Africa; ^7^Royal Hospital for Children, Glasgow, UK; ^8^Institute of Health and Wellbeing, University of Glasgow, Glasgow, UK; ^9^University of Witwatersrand, Johannesburg, South Africa; ^10^Department of Global Health, University of Washington, Seattle, WA, USA; ^11^Department of Medicine, University of Washington, Seattle, WA, USA; ^12^Department of Epidemiology, University of Washington, Seattle, WA, USA

**Keywords:** home-based counseling and testing, family-based counseling and testing, HIV testing, adolescents, intergenerational communication, family-based intervention, disclosure

## Abstract

**Introduction:**

Facility-based HIV testing does not capture many adults and children who are at risk of HIV in South Africa. This underscores the need to provide targeted, age-appropriate HIV testing for children, adolescents, and adults who are not accessing health facilities. While home-based counseling and testing has been successfully delivered in multiple settings, it also often fails to engage adolescents. To date, the full potential for testing entire families and linking them to treatment has not been evaluated.

**Methods:**

The steps to expand a successful home-based counseling and testing model to a family-based counseling and testing approach in a high HIV prevalence context in rural South Africa are described. The primary aim of this family-based model is to increase uptake of HIV testing and linkage to care for all family members, through promoting family cohesion and intergenerational communication, increasing HIV disclosure in the family, and improving antiretroviral treatment uptake, adherence, and retention. We discuss the three-phased research approach that led to the development of the family-based counseling and testing intervention.

**Results:**

The family-based intervention is designed with a maximum of five sessions, depending on the configuration of the family (young, mixed, and older families). There is an optional additional session for high-risk or vulnerable family situations. These sessions encourage HIV testing of adults, children, and adolescents and disclosure of HIV status. Families with adolescents receive an intensive training session on intergenerational communication, identified as the key causal pathway to improve testing, linkage to care, disclosure, and reduced stigma for this group. The rationale for the focus on intergenerational communication is described in relation to our formative work as well as previous literature, and potential challenges with pilot testing the intervention are explored.

**Conclusion:**

This paper maps the process for adapting a novel and largely successful home-based counseling and testing intervention for use with families. Expanding the successful home-based counseling and testing model to capture children, adolescents, and men could have significant impact, if the pilot is successful and scaled-up.

## Introduction

### Individual HIV Counseling and Testing Models

In many contexts, voluntary counseling and testing services are predominantly accessed at health-care facilities (facility-based HIV testing). However, facility-based HIV testing does not reach many adults and children (under 18 years) who are at risk of HIV in South Africa (1–5). Most adult women test through antenatal or postnatal care, but many women who are not of reproductive age, older people with high HIV prevalence (9.5%) (6–8), and men are missed by facility-based approaches. Furthermore, the children of HIV-positive women are not routinely tested through prevention of mother-to-child transmission programs (9). Despite high HIV prevalence, adolescent (defined by WHO as 10–19 years) (10) rates of testing are particularly low within facilities (11). These missed testing opportunities underscore the need to increase options to provide targeted, age-appropriate HIV testing for children and adolescents and to create opportunities for adults not accessing facility-based services to learn their serostatus.

Home-based counseling and testing involves the delivery of HIV counseling and testing by lay counselors to adults in their homes. Studies in Uganda, Kenya, Malawi, and South Africa have demonstrated that home-based counseling and testing is a highly acceptable and cost effective approach for large-scale delivery of HIV testing (12–16) and reduces opportunity costs particularly for low-income persons in rural and other under-resourced settings (16, 17). Home-based counseling and testing has also been successful in reaching first-time testers, such as couples and children (18). Increasingly, home-based counseling and testing approaches are effective in identifying and referring populations to HIV care and antiretroviral therapy (14, 19–21). Our team has developed and evaluated (22) a novel approach of home-based counseling and testing plus point-of-care CD4 results testing and facilitated referrals to HIV care in rural KwaZulu-Natal and Uganda. The results showed high (96%) uptake of testing by adults, and equally high linkage to care – at 12 months, 97% of participants eligible for treatment had linked to care – and 76% of people who were eligible, initiated treatment at 12 months (19–21).

### Family-Based HIV Counseling and Testing

While home-based counseling and testing has been successful in multiple African settings, very few models for testing entire families and linking them to treatment – the focus of this paper – have been developed or evaluated. A family-based counseling and testing approach has several potential benefits. First, it has potential to increase testing and counseling of hard to reach groups including children, adolescents, and adults missed through facility-based approaches. Second, it could efficiently link households to comprehensive HIV treatment, care, and prevention services, in particular through use of point-of-care technology. Third, and most importantly, it provides an opportunity for facilitated disclosure of HIV serostatus to family members, including children. A review of literature on home-based counseling and testing studies found that only one intervention included a group rather than individual pre-test counseling session (18); such group sessions could facilitate intra-family decision-making about HIV testing.

### Disclosure and Linkage to HIV Care and Treatment in a Family-Based Approach

Despite the inclusion of children in some home-based counseling and testing studies (14, 23–25), little attention has focused on disclosure from parents to children. Children can be affected by living with HIV-infected adults. A recent meta-analysis of demographic and health survey data from 23 countries across sub-Saharan Africa (26) demonstrated that the number of children living in households with tested, HIV-infected adults exceeded 10%; in some countries this rate was as high as 36%. Most of these children are living with parents, often mothers, who are infected. Thus, the challenge and opportunity is to design effective family interventions to support HIV testing and disclosure, which strengthens the family and supports health awareness and prevention among children and adolescents.

While other groups have conducted home-based counseling and testing of entire households, only one intervention provided support for general disclosure (14), although most encouraged couples testing, which involved disclosure (13–15, 18, 25, 27, 28). Our home-based counseling and testing studies showed that when offered, 95% of couples agreed to disclose their results to each other (19, 20). Beyond the one study which facilitated family-wide testing, no home-based counseling and testing studies could be found that describe strategies for dealing with disclosure after HIV testing, including family-based follow-up, facilitated family discussions to share information or encourage disclosure, or provision of tools to assist families dealing with the implications of HIV and AIDS (12, 25). Increased disclosure has been shown to have several benefits, including improved social support and family cohesion (29); less stigma and secrecy (30, 31); improved parent–child relationships and lower emotional difficulties in HIV affected children (32); lowered maternal depression and anxiety in parental figures (30, 31, 33); and improved compliance with health care and response to treatment for adults living with HIV (29). Thus, a family-based counseling and testing approach could provide an opportunity to encourage disclosure within families, especially between parents and children and, in particular, provide parents with the skills they require for disclosure to their children (34–38).

Early HIV testing, effective linkage to HIV care, and early antiretroviral therapy initiation have implications for prevention because, in addition to reducing morbidity and mortality (39, 40), they reduce infectiousness and, therefore, onward transmission of the virus (41–43). HIV care and treatment programs continue to utilize an individual, clinic-based approach that does not acknowledge that families are the first line in HIV prevention and the provision of support to HIV-positive family members. Children who know their status adhere better to antiretroviral therapy and are more likely to participate actively in health care (44). Similarly, adults with social support or a treatment supporter (person who helps a patient adhere to antiretroviral therapy) are more likely to adhere to treatment (45, 46). A review of family-based approaches to pediatric antiretroviral therapy has shown that the approach is very effective, with better treatment enrollment, adherence, retention, and follow up (47). Parents, in-laws, and other relatives have varying degrees of influence on decisions regarding HIV testing, disclosure, and drug treatment and adherence for children (48) and young people. Testing all family members enables the identification of multiple individuals potentially at risk (49) and could not only contribute to greater social support, pill-taking, and clinic visit adherence among HIV-positive family members on antiretroviral therapy but also prevention awareness and risk reduction (17, 23, 50).

A family-based counseling and testing approach could also address the structural factors that impact HIV transmission and infection and provide a context for more effective and sustained prevention and support. Family-centered care refers to comprehensive, one-stop HIV-prevention care and treatment offered to the family. Family-centered services, including testing and linkage to care, has mostly occurred in prevention of mother-to-child transmission settings (51–53) and has increased case finding of women and children and uptake of treatment services (52). Family-based counseling and testing could build on the family-centered prevention of mother-to-child transmission model by reducing the opportunity costs of seeking facility-based care, reducing the stigma and responsibility that clinic-identified HIV-positive family members may feel, and encouraging a more family-focused and shared response to HIV and AIDS (49, 54).

This paper outlines the process for adapting and expanding our successful home-based counseling and testing model to develop a low-intensity, scalable, family-based intervention in a high HIV prevalence and risk context in South Africa.

## Materials and Methods

### The Research Context

The intervention will be piloted in the Laduma Community, Lower Mpumuza of the Msunduzi Municipality, Umgungundlovu District, KwaZulu-Natal, South Africa. The Laduma Community is a rural area within the Msunduzi Municipality, which has a population of 618,536. It is situated approximately 25 km from Pietermaritzburg, the provincial capital of KwaZulu-Natal. The Msunduzi Municipality is characterized by high unemployment as illustrated by the provincial unemployment rate of 33% (55). This province also remains highly burdened by HIV with an overall prevalence of 16.9% among the general population, the highest of all South African provinces (56). Across all age categories, i.e., children (2–14 years old: 4.4%), youth (15–24 years old: 12%), and people of reproductive age (15–49 years old: 12%), KwaZulu-Natal has the highest prevalence of HIV in the country (56).

Ethics approval for this study was obtained from the Human Sciences Research Council Research Ethics Committee (REC 10/20/11/13). All participants in the qualitative formative phase provided written-informed consent (or assent with guardian permission, in the case of persons below 18 years old).

In this study, we define children as between 0–11 years old, adolescents as 12–17 years old, and adults as persons over 18 years old. These definitions align with the configurations of families and the South African legal framework, which provides that children 12 years and older can independently consent to an HIV test (57).

### Aims of the Study

The aim of this research, with funding from the National Institutes of Mental Health (1 R21 MH103066-01), is to develop a family-based counseling and testing model that provides HIV testing, counseling, and linkage to care *and* also supports all family members with disclosure, fosters intergenerational discussion about HIV, and increases support and health promotion among family members affected by HIV. The aims are threefold:

1. Develop a model for providing family-based counseling and testing through adaptation of our home-based counseling and testing model
2. Pilot test the family-based counseling and testing model for feasibility and acceptability
3. Assess the impact of family-based counseling and testing on testing and linkage to care, family disclosure and cohesion, intergenerational communication and stigma, and discrimination.

In this manuscript, we present the results of research objective one.

## Results: The Research Approach

**Figure 1:**
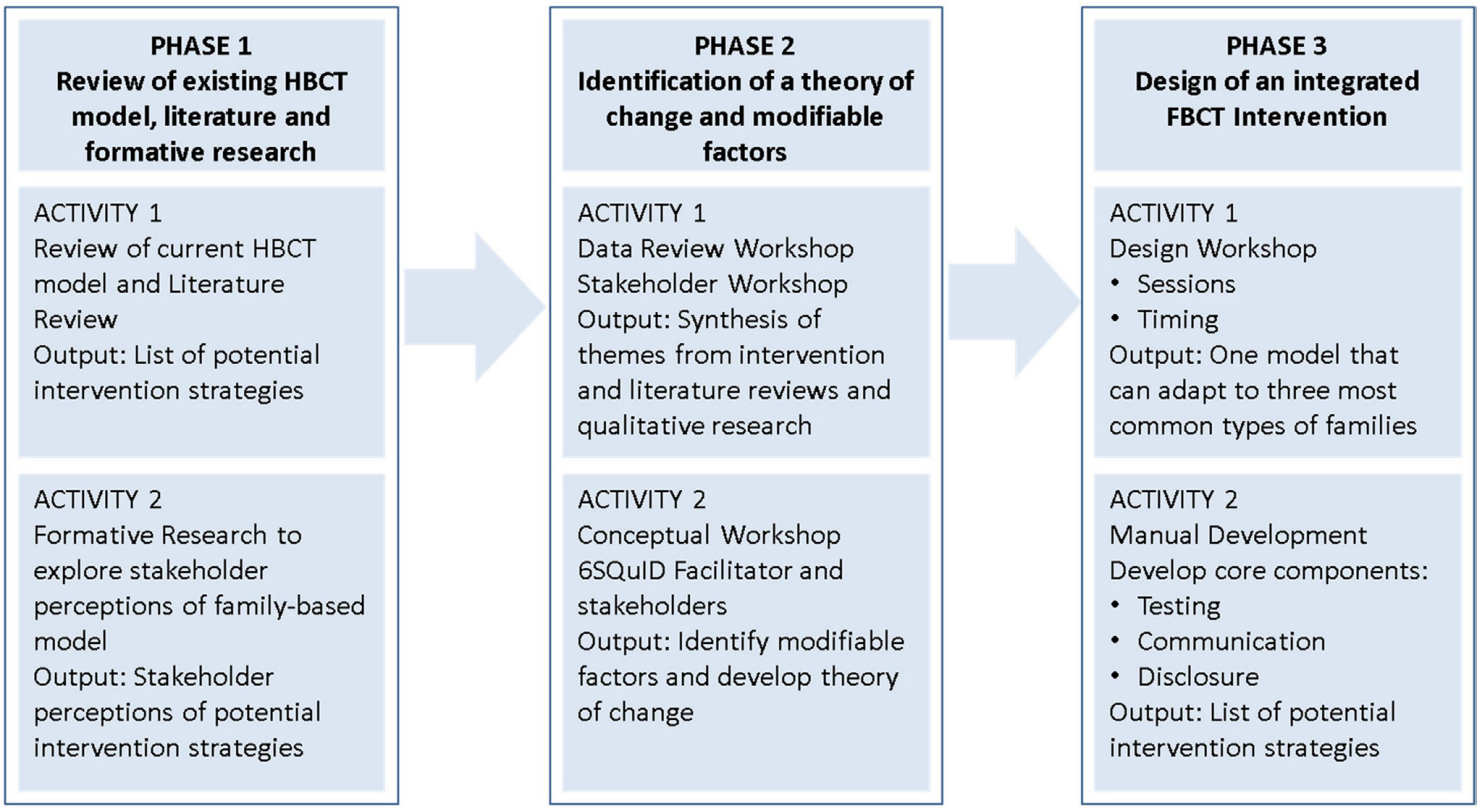
**Phases of intervention development**.

The formative research undertaken to develop the family-based counseling and testing intervention took place in three phases, outlined in Figure 1.

The intervention development process was guided by a useful pragmatic framework for developing social interventions called the *Six Steps in Quality Intervention Development* (6SQuID) model (58). This framework comprises six critical steps, namely: (1) defining and understanding the problem and its causes; (2) identifying which causal or contextual factors are malleable and have the greatest scope for change; (3) deciding how to bring about the change mechanism; (4) identifying how to deliver the change mechanism; (5) testing and adapting the intervention; and (6) collecting sufficient evidence of effectiveness to proceed to rigorous evaluation of the intervention.

The approach taken in this research was not to develop a new intervention, but instead to augment a current successful home-based counseling and testing intervention to include families. During this phase we addressed steps 1–4 of the 6SQuID model through three key activities:

1. A review of the current home-based counseling and testing model, literature, and formative research
2. Identification of a theory of change and modifiable factors
3. Design of an integrated family-based counseling and testing intervention

### Phase 1: Review of Existing Home-Based Counseling and Testing Model, Literature, and Formative Research

#### Review of Current Home-Based Counseling and Testing Model

The home-based counseling and testing model starts with community mobilization to inform and prepare adult household members over 18 years old for the study. Counseling and HIV testing is then conducted by lay counselors or enrolled nurse assistants in the home. HIV seropositive persons receive point-of-care CD4 testing at the same visit, and referrals to HIV care are made. Follow-up visits of HIV-infected persons are conducted quarterly to assess uptake of clinic visits and antiretroviral therapy initiation and to provide counseling about HIV care and antiretroviral therapy adherence. If a couple participates, they are counseled and tested separately; facilitated disclosure is provided with their permission.

Augmenting the current home-based counseling and testing model to include all family members (adults, adolescents, and children) involved two related activities: (1) a systematic review of the literature on family-based interventions to inform the content, processes, and development of suitable strategies for developing a family-based counseling and testing model and (2) formative research to explore the familial, sociocultural, and community factors that could impact the effective delivery of a family-based counseling and testing model.

#### Literature Review

We conducted a systematic review of the existing intervention literature focusing on family-based interventions in general, those that addressed HIV testing in families, including children and adolescents, and those that encouraged family disclosure and communication. Key search terms included families, sex/HIV, communication/intergenerational communication, and setting – as well as variations thereof. The preferred reporting items for systematic reviews and meta-analyses (PRISMA) approach was used to guide the review. We searched online databases (ProQuest Central, Pubmed, and EBSCO Host) and numerous additional databases and journals that were indexed within these. The search identified 23,782 articles, after duplicates were removed. After a scan of titles and abstracts, we retained 186 articles, which were separated into primary and secondary papers. Primary papers (*n* = 97) were included in the data extraction and the systematic review. Secondary papers were articles of interest that provided important context/background information for the study. Primary studies were coded according to pre-defined fields, including details of reference (title, author, year of publication), study characteristics, design, setting, outcomes, etc., which were summarized for the systematic review. To ensure consistency in coding, each article was independently coded and summarized by two researchers. Appendix Table A1 provides a summary of child and adolescent family-based interventions conducted in South Africa.

#### Formative Research

Formative research is often used to inform the design and delivery of interventions. However, this critical process is rarely reported. Substantial formative work prior to implementing interventions has been recommended by McKleroy et al. (59) and supported by others (60–62). Data collection was undertaken between September and November 2014 to establish adults, children, and adolescents needs, concerns, and perspectives of the potential family-based model. We conducted 40 in-depth interviews with 20 key informants and 20 stakeholder representatives, as well as 12 focus group discussions with male and female adolescents between 13–18 years old (*N* = 77). Participants were purposively sampled. Interviews and focus groups were audio-recorded, transcribed, translated, and thematically analyzed (63) in multiple iterations by two researchers.

### Phase 2: Identification of a Theory of Change and Modifiable Factors

Once the qualitative data and review outputs were available, the research team convened in a series of workshops to synthesize results from the systematic review and formative research to develop a conceptual framework and elucidate the theory of change for the family-based counseling and testing model. The involvement of community and implementation stakeholders in these workshops, recognized that intervention development is best approached through multidisciplinary stakeholder teams including researchers, practitioners, the affected population, and policy makers (58).

The first workshop focused on data review and presented the results of the qualitative research to the investigator group. The results are under review elsewhere [Gillespie et al. (2016), Knight et al. (2016), and Ngcobo et al. (2016) – abstracts submitted to International AIDS Society (IAS) Conference, 2016]. The results illustrated that a family-based approach was in principle highly acceptable but that stakeholders expressed concerns regarding testing adolescents with their caregivers, intergenerational communication on subjects such as sex, sexuality, and, thus, HIV. Parental figures expressed a lack of confidence, knowledge, and skills in dealing with this issue with children and adolescents. The outcome of this workshop and review of the literature led to a decision to conduct a second workshop, facilitated by an expert consultant, to better define the theory of change for the intervention and to clarify which contextual factors were likely to have the greatest impact in reaching our study outcomes.

This second workshop with a group of diverse stakeholders took place over 2 days and attempted to integrate the results from the literature review and the formative data in order to develop a clearer understanding of which causal or contextual factors had the potential to impact the family-based counseling and testing model outcomes and which intervention activities would have the greatest scope for change. During these two workshops, steps 1–4 of the 6SQuID framework were addressed (58). The third and fourth workshops involved the investigators designing the model and refining intervention activities and tools.

**Figure 2:**
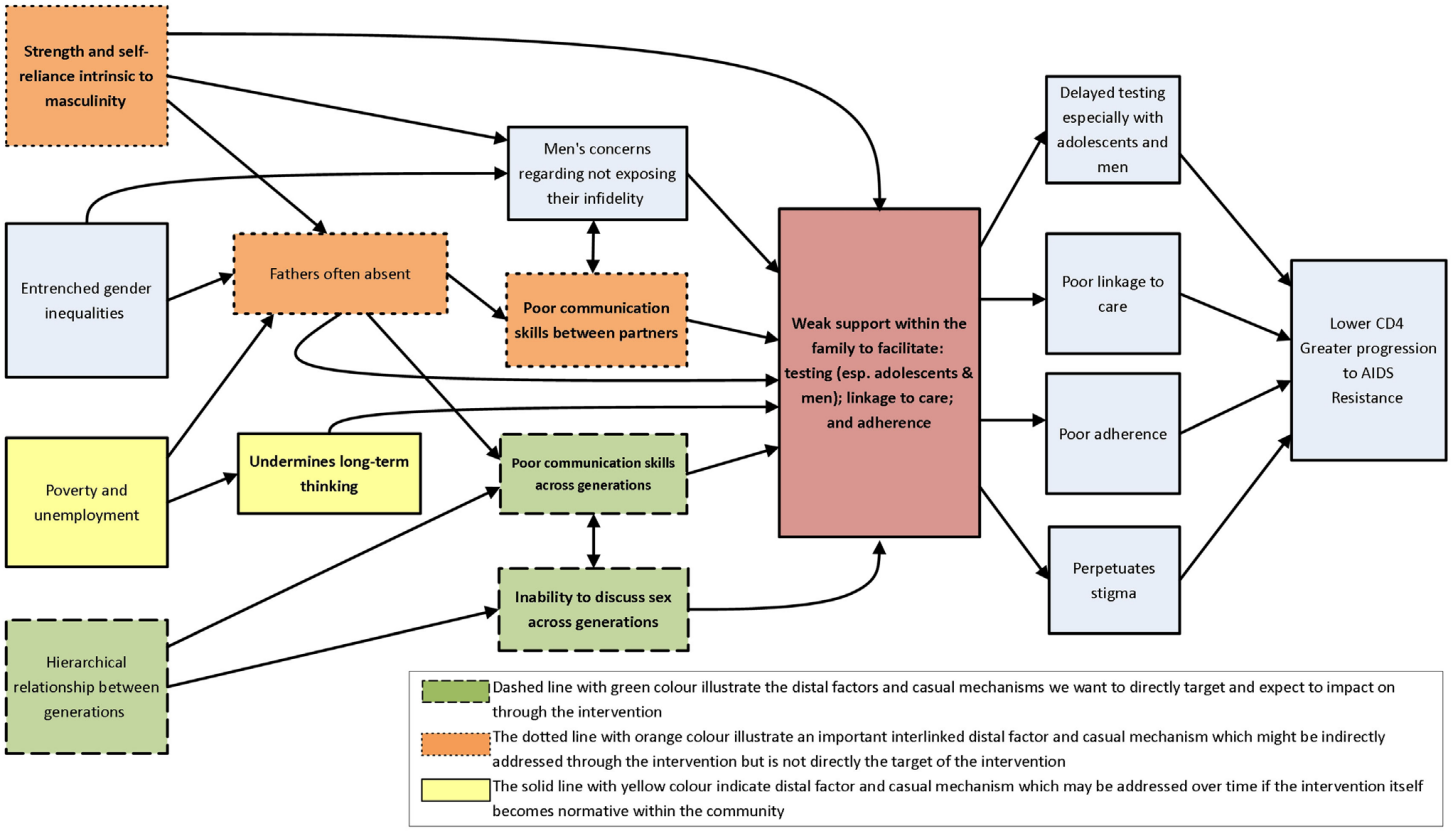
**Causal pathways perpetuating weak support within the family to test for HIV, link to care, and support adherence**.

As illustrated in Figure 2 (see below) at these workshops, the main problem we identified was that families had weak support, which led to poor HIV testing, linkage to care, and adherence to treatment. After clarifying the problem, we made efforts to understand its causes (the immediate and underlying influences). Several causal pathways were identified, namely, strength and self-reliance being intrinsic to masculinity, entrenched gender inequalities, poverty and unemployment, hierarchical relationships between generations, absent fathers, men’s anxieties regarding exposing infidelity, poor communication skills between partners, poor intergenerational communication skills, and inability to discuss sex across generations (depicted on the left in Figure 2). This critical step of representing, diagrammatically, the causal pathways leading to the problem was essential to carefully consider how best to intervene to improve outcomes. These outcomes (depicted on the right in Figure 2) included delayed testing (especially among adolescents and men), poor linkage to care, poor adherence, and stigma. Without intervention, these causal and contextual factors ultimately contribute to lower CD4 counts, greater progression to AIDS, and worse treatment outcomes.

As a next step, efforts were made to clarify which causal or contextual factors were modifiable through this intervention and would have the greatest scope for change. The different colors of the boxes on the left in Figure 2 represent different potential pathways to change. During this step, we carefully considered which of the various pathways would be most amenable to change, which changes would have the most effect, and who would be most affected by them.

Three important modifiable causal and contextual factors were identified, which could form the intervention target. These included hierarchical relationships between generations, inability to discuss sex across generations, and poor communication skills across generations. The formative work and the literature review identified great value in parent–child communication in mitigating high-risk behavior (64). We recognized that adolescents are a key target population for HIV-prevention interventions given their high risk; in South Africa, the incidence among adolescents was higher than for any other age category, at 1.49% (56).

Our formative research, systematic review, and the consultative workshop elucidated that intergenerational communication was the most modifiable causal pathway for this family-based intervention and the one with the greatest potential direct impact on our research outcomes. Given the limited time-frames of this study, the team recognized that, while “upstream” structural issues (such as gender inequalities and masculinity) and couple relationships were important distal factors that could impact family-based counseling and testing, these did not represent the most modifiable pathways for this intervention. Our team is also involved in conducting separate studies that directly address couples (1R02MH086346-01A) and male involvement in testing and treatment (1R01MH105534-01A1).

This family-based intervention will use a theory of change informed by a theoretical framework to effect change.

#### Theory of Change

Hierarchical relationships between generations, inability to discuss sex across generations, and poor communication skills across generations were three important factors that could impact on the primary outcomes of this study that is improved uptake of HIV testing and linkage to care for all family members, improved discussion and disclosure of HIV status among families, improved family cohesion, and improved antiretroviral therapy adherence and retention. Our intervention aims to address these outcomes through changing participants’ knowledge, perceptions of risks and benefits, awareness, social norms, skills, self-efficacy, and intentions regarding testing, treatment, and disclosure. This will occur over a series of sessions with families, through engaging them in counseling, information, and support activities, many of which use modeling. The study will also identify a change agent in the family who could act as a catalyst for change by helping the family transform itself. The change agent will be identified in the first session and help the counselor lead discussions in the remainder of the sessions.

In developing a family-based home-based counseling and testing model, we drew on Ewart’s social action theory (SAT) (65). The approach has been used successfully in couples and family-based interventions addressing mental illness (66) and interventions to improve HIV medication adherence (67). SAT recognizes the interwoven relationship between the individual, family, and community factors in determining uptake of interventions to promote self-protective behavior. Parent–child relationships may facilitate or impede disclosure and discussion about HIV and AIDS, provide helpful action plans or role models for communication and disclosure, and foster strategies for modifying “scripts” that keep HIV status as a family secret and impede disclosure. We hypothesize that, where whole families are tested and opportunities for family discussions and HIV disclosure are encouraged, HIV testing and antiretroviral therapy uptake may be improved, leading to greater social support for HIV-positive individuals, improved adherence to antiretroviral therapy, and prevention awareness and risk reduction in the family (17, 50).

### Phase 3: Design of an Integrated Family-Based Counseling and Testing Intervention

The final step in this phase involved identifying how best to deliver the change mechanism through intervention design. Operationalizing the intervention was an iterative process that included results from the systematic review and formative research and refinement during the stakeholder conceptual workshop. Investigators drew on these prior steps in two intervention design workshops that focused on refining the family-based counseling and testing model, process, activities, and tools.

The intervention will be delivered by trained counselors/facilitators. Counselors and implementation staff were included in all aspects of the process drawing on their knowledge and experience to ensure the design of a feasible intervention that could work in this context. We also consulted with experts in child and adolescent development to produce and adapt tools and materials to test children, adolescents, and adults for HIV to encourage disclosure and to improve intergenerational communication.

**Figure 3:**
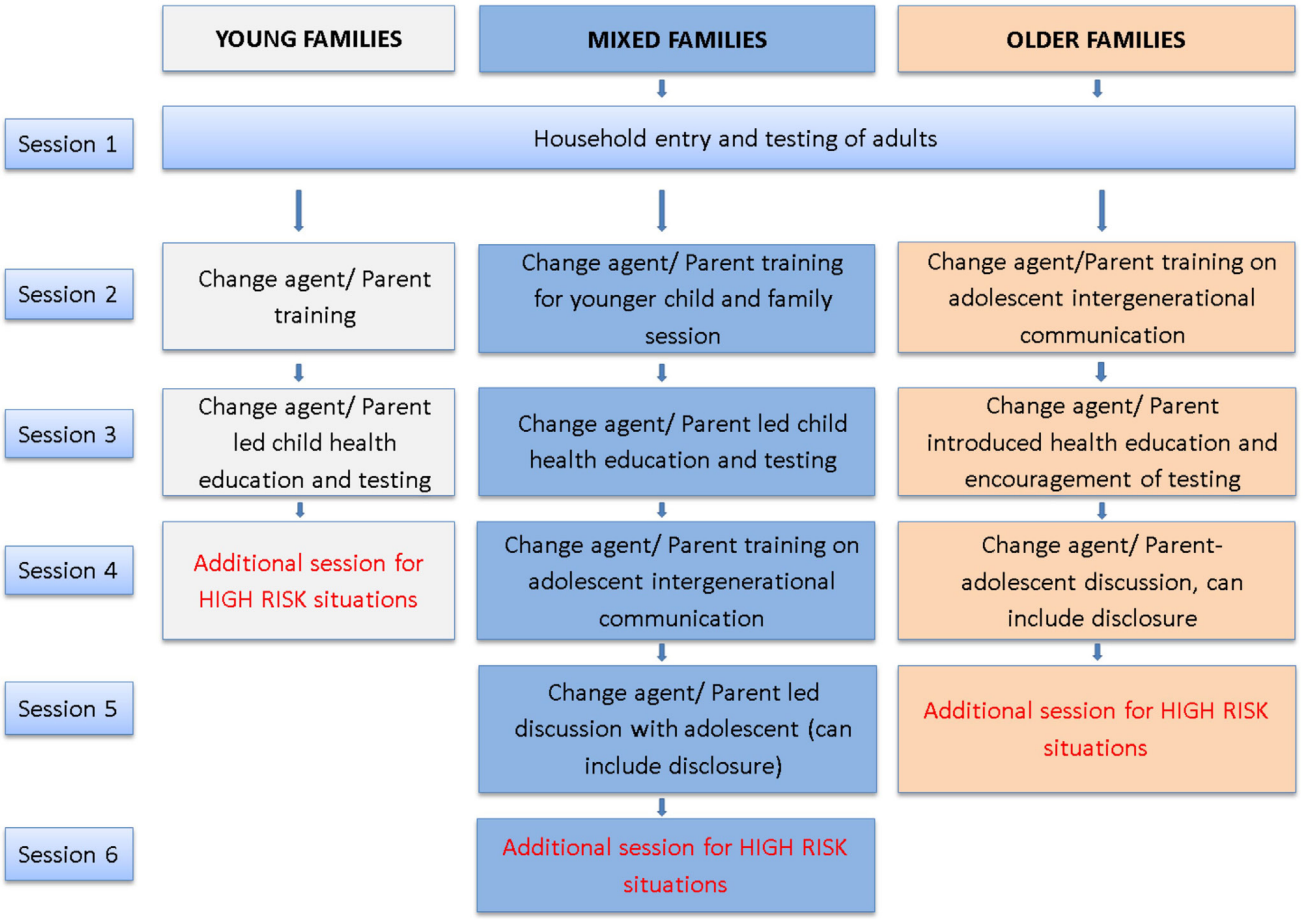
**Flowchart of the family-based counseling and testing intervention for different configurations of family**.

The proposed family-based counseling and testing behavioral intervention consists of up to five sessions delivered within the household, plus an optional session for high-risk or vulnerable family situations (see Figure 3 below). The intervention is expected to cater to three configurations of families: (1) young families have adults and children, where all resident children are 11 years or younger; (2) mixed families have adults and children, where some children are 11 or younger and some are 12–17 years old (adolescents); and (3) older families comprising adults and adolescents (children 12–17 years) with no young children resident. Steps on how the intervention will be approached with each type of family are described in detail below.

#### Household Entry and Testing of Adults

On a first visit to the household, all families will receive an introduction to the study. This facilitator-led session will identify the family configuration (through various tools and activities) and identify and recruit the change agent(s) who will cofacilitate future sessions with the facilitator/counselor. There may be one change agent or a dyad who could take on the change agent role (e.g., parents, sisters, mother, and grandmother). Change agent(s) will be selected based on pre-defined criteria, namely: they must be available for all sessions of the intervention; preferably older than 18 years; willing to be a change agent; able to facilitate training; and must have good relations within the family. Two sets of activities will be completed in this session. The *Family Tree Activity* (38) will be a joint family activity used to identify all members of the family to assist in categorization of type of family (young, mixed, older), and to identity through discussions potential change agent(s). Participation in this activity may also foster a sense of family belonging. The *Let’s Test Activity* for all adult family members is cofacilitated by the counselor and the identified change agent. This session follows the joint family session and provides information about HIV risk behaviors and encourages HIV testing. The facilitator will then conduct individual or couples pre-test counseling in a private space, test family members who agreed to test, conduct detailed post-test counseling for the tested members, and offer appropriate referrals. Family members will be encouraged to test as a group or as couples. Formative data indicate that generally, respondents perceived the family-based counseling and testing approach as feasible and acceptable, and positively considered receiving the intervention at home instead of at health-care facilities.

A PIMA point-of-care CD4 test will be conducted for all HIV-positive adults to facilitate linkages to care. Adult family members who have tested will be counseled on their results and, if necessary, referred to local health-care facilities. The benefits of disclosure and the importance of treatment and care will be emphasized in discussions, and adult family members will be encouraged to share their results with other adult family members. Our formative research identified some concern about inadvertent disclosure of HIV test results to family members and the community. The intervention is sensitive to this concern and allows for multiple permutations in terms of disclosure (within couples or the larger family group and at different time points). However, disclosure was also identified as a potential positive outcome of the intervention in that it may foster supportive family relationships and facilitate family cohesion.

#### Change Agent Training

This session is configured differently dependent on family composition. In younger and mixed families, this will involve training the change agent/s to cofacilitate the family health education and testing session with children. Change agents will be trained by counselors/facilitators on several activities or materials, namely, the body map, health promotion cards, and the *Let’s Test* poster for children (38). The *Let’s Test* activity intends to provide age-appropriate health and illness information to young children in an engaging and informative way. It is a story telling aid that facilitates discussion on health and illness between change agents/parents and their children. Health promotion cards aim to reinforce positive living messages. The activity is supported by a set of 20 playing cards (10 pairs). The cards include images and health promotion messages. A second facilitator will test any adults who did not test during the first session and encourage disclosure among those who did not disclose.

For older and mixed families (during sessions two and four respectively), change agents/parents will undergo training on intergenerational communication, the key change mechanism in the intervention for addressing issues with adolescents. Our formative research revealed that adolescents are open to communicating with their parents, but that several barriers exist. Many adolescents discussed difficulty initiating conversations but parents also noted barriers to intergenerational communication, such as cultural taboos about discussing sex. Through the formative work and stakeholder consultations, we recognized that a focus on intergenerational communication that would support parents/caregivers to develop general communication skills with adolescents in the household and equip them to deal with sensitive issues, including HIV and disclosure, would be the best causal pathway to effect change in the outcome measures.

When designing the intergenerational communication session, we incorporated common elements of successful intergenerational communication studies (68), including the *Let’s Talk* intervention, which has been culturally adapted for South Africa (61). The interactive 90-min session addresses:

1. *Communication skills*: The counselor will discuss various strategies to improve both the quality and quantity of communication with adolescents, including developing listening skills, such as open-ended questions, active/reflective listening, providing verbal support and non-judgmental responses, and rephrasing. Change agents/parents will be provided with the opportunity to role-play some of these critical skills.
2. *Fostering positive relationships between parents and adolescents through identifying and positively reinforcing good behaviors*. Vignettes will be used to help change agents identify examples of opportunities for positive reinforcement and offer an example of a positive parenting strategy.
3. *Talking about sensitive topics, including HIV*: The counselor will describe strategies to discuss sensitive topics with adolescents, using role-plays and vignettes. This component aims to identify social norms, create awareness, and provide parents with skills to communicate difficult issues.

Homework activities tailored to different age categories of adolescents are designed to allow parents the opportunity to implement what they have learnt with their adolescents outside of the training environment. Such homework exercises are supported by research evidence that indicates these activities can increase family communication about sexual issues and successfully delay sexual debut among early adolescents (69).

Finally, the change agent/parent will also be trained on how to encourage their adolescents to test for various health conditions including HIV, using *The Let’s Test* activity for adolescents.

#### Testing of Children and Adolescents

For young and mixed families, at the next session with the family, the change agent will lead a session on child health education and testing for all children 11 years and younger, cofacilitated by the counselor. Children will be tested for HIV with their parent’s consent and their assent, according to the ethical–legal framework for HIV counseling and testing in South Africa (57). Children over 5 years old will receive point-of-care CD4 testing, and all HIV-positive children will be referred to local health-care facilities for clinical assessment, care, and treatment. If a child under 5 years old tests HIV-positive, the parent and child will be referred to the local health-care facility for CD4 testing, clinical assessment care, and treatment. Information about the importance of antiretroviral therapy, accessing timely treatment, and the value of social support will be provided to parents of all tested children. Disclosure of the child’s HIV status and the process by which this may happen will be discussed with the parent/guardian and facilitated using the *Disclosure Safety Hand* activity (38). Previous research has identified that one of the key barriers in disclosure to children is concern that they may disclose to others (34, 38). For this reason, this is an interactive activity for facilitated disclosure with children that encourages them to disclose to and have discussions about their (or their parent’s/family member’s) status with those trusted people specified on the hand only. The facilitator will train the change agent on the *Disclosure Safety Hand*.

For mixed and older families, within 2 weeks of the intergenerational communication training of the change agent/parent, a health education session and HIV testing session for adolescents will be conducted. This *Let’s Test* session will be led by the parent/change agent and facilitator.

Adolescents aged 12 years and older are permitted to consent independently for HIV testing (57). The session will emphasize adolescents’ rights to privacy and confidentiality of the testing process and their test result, including any information that may emerge in counseling. However, the benefits of disclosure and the possibilities of family support will be discussed during the pre-test information session. Our family-based counseling and testing intervention strongly focuses on supporting disclosure within families and facilitating open and accurate intergenerational communication on sex and HIV. Counseling and testing will be provided to all adolescents in the household as a group. Age-appropriate pre-and post-test counseling messaging will be provided to adolescents in groupings of their preference (individually, pairs, or groups), although they will be encouraged to test as a pair or group rather than individual for mutual support. Adolescents who are HIV-negative will be provided with age-appropriate risk reduction and repeat testing counseling messages. A PIMA point-of-care CD4 test will be conducted in the household in order to facilitate linkages to care. The benefits of disclosure and the importance of treatment and care will be emphasized.

#### Follow-up for High-Risk Families

The final session is an optional session for high-risk situations available to all three family configurations. High-risk targets will include any households with HIV-positive children aged 0–11 years old; HIV-positive adolescents where disclosure is an issue; individuals with suicidal ideation or a crisis as a result of testing situation; and households where there are other important risks, such as conflict, domestic violence, substance abuse, serious mental health issues, among others.

Where a child has tested HIV-positive on previous visits, we will use this session to ensure that all referrals for follow-up health care, treatment, and support were addressed. If referrals have not been taken up, we will explore the reasons for this and provide strategies and support for how this could be addressed. We will also provide additional communication skills to the parent/caregiver on how to observe and engage with the emotions and feelings of the HIV-positive child.

In the case of adolescents who test HIV-positive and have not disclosed their status, this follow-up session will provide an additional opportunity for adolescents and parents/caregivers to discuss any barriers that may be preventing them from doing so. Staff will address this through facilitated discussions between generations, reinforcing some of the positive parent communication skills addressed in previous sessions. Parents/caregivers will be encouraged to take the lead in addressing these issues. Follow-up education and support on the benefits of disclosure, treatment, and adherence will be provided to children and adolescents.

This follow-up session also allows us to make a final assessment of family needs and necessary referrals to appropriate services. As we work with households, we may encounter families who may be living in extreme poverty with low food security, or where children are not schooling or we may identify medical needs in children. In all these instances, depending on the issues, referral will be made to local primary health-care clinic, education, and social welfare groups, non-governmental and community-based groups working in study communities, and with whom we have referral networks.

Over and above these situations, we may also encounter a range of risk situations in these families. These may include relationship problems/conflicts, domestic violence, feelings of hopelessness, and suicidal thoughts without serious intent or plans, as well as serious mental health concerns. We will have mechanisms to promptly identify and respond to these through our fieldwork, and appropriate referrals through our networks described above will be made. Management of severe mental health issues and suicide ideation will include referrals to a psychiatric nurse, clinical psychologist, or psychiatrist. As with previous studies, we keep careful documentation of all referrals and any adverse events and report them annually at IRB recertification.

## Discussion

This paper described the three phases of intervention development and detailed the various components of the family-based counseling and testing intervention. We end with a discussion of a few issues that may be challenging, as we implement the intervention.

This intervention aims to test all family members for HIV, encourage disclosure, and facilitate linkage to care. In the main, these aims are associated with positive outcomes, such as improved prevention, care, and treatment, and better social support from friends and families. However, some studies have documented potential negative consequences of HIV testing and disclosure. In relation to children younger than 11 years old, key concerns may include that children are too young, they may endure negative emotional consequences as a result of disclosure of their own or their parents’ HIV status, and they may inadvertently or otherwise disclose to others outside the family (34, 38, 70). With adults, disclosure of a positive HIV status may also result in disruptions to relationships with families and communities, social isolation and ostracism, abuse, violence, divorce, and rejection (71). In cases where facilitators identify the potential for negative consequences, an additional session (for high-risk situations) will be conducted.

The key to unlocking this intervention rests on communication between adults in the household and adolescents. A primary challenge with this is that many of the communities targeted for HIV-prevention interventions, including ours, are influenced by traditional mores and values, which view sex as a taboo subject that should not be openly discussed (72, 73). Previous research in a South African rural Zulu community revealed that discussions about sex between younger and older people are largely forbidden, and that, when it does occur, such discussion is obscured by the use of polite language, euphemisms, and gestures (72). A qualitative study with parents and adolescents in Cape Town also found taboos challenged family interactions about sex (73). Further, an absence of parent–adolescent communication about sex reinforced taboos about discussing sex (72, 73).

In such contexts, sex is traditionally only discussed with adolescents when they reach puberty or in preparation for marriage, as rites of passage by an extended family member of the same gender, rather than from parents or caregivers (72, 74, 75). However, these traditions have largely disintegrated, leaving an important gap in terms of the sex education of youth (72, 74). Nevertheless researchers in India, which is characterized by similar conservative cultural mores regarding adolescent sex, found that parents were open to discussions with their children and that training may help mitigate some of the discomfort in discussing sensitive issues (76). This is also supported by evidence from our formative research with adults. This suggests that cultural prohibitions are not unchangeable and may be addressed through appropriately designed interventions that promote open and clear communication about sensitive issues, including HIV and AIDS (76). Our intergenerational session emphasizes parents’ critical role in informing their adolescents about sex, including about their perspectives and values. Resonating with our formative research, which found that adolescents are receptive to communication with their parents, research also indicates that adolescents want to discuss sex with their parents but that parents need improved communication skills (77, 78).

In contrast to this conservative sociocultural milieu, South Africa has a progressive legal framework which enables adolescents 12–17 years old to access a range of sexual and reproductive health services including contraceptives, treatment for sexually transmitted infections, and testing for HIV (79). Related to their right to independently consent to various health services, adolescents have a right to privacy of their test results (80). However, this right to privacy is limited by mandatory reporting obligations, which require that all sexual offenses against a child must be reported (80). These legal provisions serve as both barriers and facilitators to the implementation of the family-based counseling and testing intervention.

Since HIV testing and disclosure occur as part of a research study, all children below 18 years old require parental consent to participate (57). As such, parents or guardians who provide consent for their children’s participation in the study may reasonably expect to be informed of their child’s personal health information (57). However, the South African legal framework provides that adolescents 12–17 years old have the right to privacy regarding certain therapeutic health interventions that form part of the HIV-prevention study, and, therefore, researchers cannot disclose such information to parents/guardians (80). These limits are spelled out in the parental informed consent forms for children’s participation in this study. However, given that a central aim of the study is disclosure of HIV test results, trained counselors will provide support, including practice and feedback through role-playing, and encouragement to adolescents to disclose their HIV status to a family member or trusted adult.

On the other hand, adolescents who are engaged in behaviors that make them vulnerable to HIV infection may be reluctant to discuss these behaviors with their parents and may have legitimate concerns regarding negative reactions from their parents. Therefore, protecting adolescent rights to privacy may encourage them to test for HIV, but create challenges for disclosure.

A further limit to adolescents’ privacy rights is the requirement for mandatory reporting of all sexual offenses committed against children, including consensual sexual activity (81). However, recent amendments to the Sexual Offences Act, which decriminalize underage consensual sex, narrows this limit to privacy by restricting the offenses that need to be reported. The impact on adolescents in this study, is that their privacy will be limited only in circumstances where, “the activity was non-consensual; the younger participant was 12–15 years old and the older participant 16–17 years, and the age difference between them was more than 2 years at the time of the act; and the younger participant was 12–15 years old and their partner was an adult” (79). Such limits to confidentiality will be spelled out to parents and adolescents during the informed consent process.

## Conclusion

Expanding our successful home-based counseling and testing model to a comprehensive family-based model could have significant impact in our high HIV prevalence context. Testing families could increase the identification of HIV-positive children before they become sick enabling early linkage to care and for them to gain larger and longer benefits from antiretroviral therapy. HIV testing of all family members, disclosure, and linkage to care are critical to ensuring that infected family members are enrolled into care timeously in order to achieve positive treatment outcomes (21, 82). Our approach treats the family as a social environment (not just a location for service delivery), through which HIV prevention, treatment, adherence, and support could be achieved (50). Our intervention targets families and includes components to improve uptake of testing among children and adolescents, facilitates HIV disclosure and support among families, and encourage intergenerational communication, including regarding sexual risks for HIV. We address the complexities of HIV disclosure and communication between family members through the provision of various tools and strategies (12, 25).

In the next phase of the study, we plan to address study Aims 2 and 3, which align with Steps 5 of the 6SQuID the model – testing and refining the intervention – and Step 6 – collecting sufficient evidence of effectiveness to proceed to rigorous evaluation. In this next phase, the intervention will be piloted with 50 families, using a combination of quantitative and qualitative methods to evaluate the feasibility and acceptability of the model (Aims 2 and 3). This Phase will also help identify any further unanticipated implementation challenges.

## Author Contributions

HR, ZE, and TR conceptualized the paper. HR and ZE developed the first draft. TR, DW, LK, RB, and CC made comments/inputs on the manuscript. HR and LK analyzed formative qualitative data. DW facilitated the consultation on development of the model.

## Conflict of Interest Statement

The authors declare that the research was conducted in the absence of any commercial or financial relationships that could be construed as a potential conflict of interest.

## Appendix

**Table A1:** **Summary of child and adolescent family-based interventions conducted in South Africa**.

Reference	Focus	Ages of participants	Intervention description
Bhana et al. (83)	To support families in promoting the health and psychosocial well-being of youth living with perinatal HIV infection	Children: 10–14 years old Caregivers: Age is not indicated	Six sessions over 3 months Session curriculum involved HIV-infected youth and primary caregiver and other family members. Family group activities and separate parent and child group activities. Cartoon-based storyline was used
Bogart et al. (61)	Parent–child communication about HIV and sexual health and parent condom use self-efficacy and behavior	Parents of 11–15 year olds	Five weekly 2-h group sessions with parents of youth. Topics included building relationship with your child, talking about sensitive topics, talking about HIV and condoms and building assertiveness skills
Rochat et al. (38) (Amagugu)	Development of a family-centered, structured intervention to support mothers to disclose their HIV status to their HIV-negative school aged children	Children: 6–9 years Mothers	Six sessions using an intervention package that comprised printed materials, therapeutic tools, and child-friendly activities and games to support age-appropriate maternal HIV disclosure. (i) Either a lay counselor or community health worker (CHW) offers assistance and trains the mother toward disclosure, (ii) the mother undertakes disclosure with the child on her own, (iii) the mother takes the child to the clinic independently, and (iv) completes a care plan and custody plan without the counselor being present.
Bell et al. (84) (CHAMPSA)	To test the effectiveness of the CHAMP among black South Africans in South Africa. The CHAMPSA intervention targeted HIV risk behaviors by strengthening family relationship processes as well as targeting peer influences through enhancing social problem solving and peer negotiation skills for youths	Children: 9–13 years Adult caregivers	Intervention was delivered on weekends by community caregivers trained as facilitators Intervention was annualized and a step-by-step facilitator manual was developed to guide the facilitators. The manual introduced skills through dramatic depiction in a cartoon-based storyline
Bhana et al. (85)	Participatory adult education principles, participatory cartoon-based narrative methods to deliver its content	Adults – age is not specified	Delivered through a series of manual-based sessions to groups of families with pre-adolescent children and evaluated using a treatment vs. a no-treatment repeated measures design. Small groups were used to deliver the intervention. An open-ended participatory approach was used
